# Book Review

**Published:** 1923-03

**Authors:** 


### Reviews of Books.

**The Physical Examination of the Chest.** By JAMES CROCKET, M.D., D.P.H. Pp. viii., 254. London: H. K. Lewis & Co. Ltd. 1922. Price 9s. net.—This book should be of great use to the advanced student and also to the practitioner. The author lays great stress on the necessity for general observation of the patient before examination of any particular system, and in these days of instrumental aid in diagnosis this is a point which should be very necessarily emphasised. The chapters of the book follow the methods of examination. Inspection, palpation, percussion, and auscultation are dealt with exhaustively, and they are preceded by a chapter on the anatomy of the thorax. An explanation of all the signs is given, which is a very good method of enforcing them on the memory and also gives interest to the reader. The chapters on X-rays in diagnosis and the examination of the larynx are a necessary adjunct to the examination of the chest. The subject-matter is expressed simply and clearly and the illustrations are excellent.

**Artificial Limbs and Amputation Stumps.** By MUIRHEAD LITTLE, F.R.C.S. Eng. Pp. vii., 319. London: H. K. Lewis & Co. Ltd. 1922. Price 25s.—The author of this book has had an almost unrivalled experience in the subject, having being engaged as principal surgeon at the Roehampton Hospital, which was devoted to limb-fitting work during the War, and under the Pensions Ministry. During this time about 25,000 cases of amputation have been under his care. Two features of the book greatly enhance its value, the first being that the author has confined himself to practical consideration and made his work of comparatively small and convenient size; the second is that he has delayed publication until sufficient time has elapsed to allow mature judgment and experience to be brought to bear on the various types of limbs which are now available. After a short historical note, four chapters are devoted to a consideration of stumps and the special modifications of cinema plastic surgery, the relations of the natural joints to those of the artificial limb, and the consideration of the comparative usefulness of artificial arms and legs. The bulk of the book consists of a detailed description of artificial limbs suitable for all types of amputation, including those official patterns approved and standardised by the Pensions Ministry. As a book of reference this work is invaluable, and the numerous and clear figures and diagrams make it easy to follow the text. The great drawback to the book is the presentation of so many alternative appliances without clear indication as to the circumstances which should guide in choosing between one and another.

**A Text-Book of the Practice of Medicine.** By Various Authors. Edited by FREDERICK W. PRICE, M.D., F.R.C.P. Pp. xxxvi., 1,753. London: Henry Frowde and Hodder and Stoughton. 1922. Price 35s. net.—The Editor's aim has been to include within one volume a concise survey of the whole field of medicine. He does not think that any one man is capable of writing such a book, and for this reason he has invited a number of his colleagues, representing nearly all the London Medical Schools, to join him in his venture. The result is a very good book. In his preface Dr. Price says that his earnest hope is that the book may be considered a credit to the London School of Medicine, and this is indeed exactly what it is. It is reassuring to find it characterised by those virtues on which the London School has securely founded its fame. A respect for facts, and particularly for clinical facts, is apparent in most of the contributions. Moreover, the writers have not forgotten that the purpose of a teacher of medicine is to instruct students in the art of treating patients. Some of the newer subjects are allowed rather more notice than they deserve. For example, more space is accorded to trench fever than to measles, more to lethargic encephalitis than to tetanus. On the other hand, some of the articles are remarkably compact. The whole section on diseases of the endocrine glands, including an excellent little introduction on the relation of those glands to the sympathetic nervous system, occupies only 26 pages, yet there is not much in the way of established fact that has been left out (although a word on the subject of vomiting in Graves's disease might not be amiss). The Editor's zeal for polygraphic and electrocardiographic records has rather outrun his discretion, otherwise there are very few illustrations. But the book is printed in a good type, and the use of a thin paper has kept the bulk within convenient limits. Though we must confess to looking back with regret on the passing of the " one-man " text-book of medicine, it must be admitted that it would be difficult for any one physician to achieve such a task at the present day. Even Sir William Osler's famous book did not include a section on mental disease, as this composite work does. Whether it is regarded as a compendious text-book for students or a concise book of reference for practitioners, this volume is sure of a warm welcome and a successful career. We doubt whether there is a better in the English language.

**A Practical Handbook on the Diseases of Children.** By BERNARD MYERS, C.M.G., M.D., M.R.C.P. Pp. xvi., 548. London: H. K. Lewis & Co. Ltd. 1922. Price 21s. net.—This work, Dr. Myers states in his preface, embodies twenty-one years' experience of practice in the diseases of children. The author, in view of the rapid advances made in certain special medical subjects, has entrusted the article on " the Biochemistry of Children's Diseases " to Dr. R. L. Mackenzie Wallis, Dr. John Eyre writes the article on " the Use of Sera and Vaccines," Mr. A. Rocyn Jones is responsible for all surgical references to Orthopædics, Col. E. G. French has written " on Syphilis in Children," Mr. Montague Hopson on " the Teeth," Sir. W. M. Bayliss " the Physiology of Digestion," Dr. H. G. Adamson has revised the section of " Diseases of the Skin," Dr. T. Joeker is partly responsible for the description of " the Normal Blood in Children," and also has contributed " the Laboratory Methods of Diagnosing Tuberculosis." The early chapters of the book are distinctly good and full of information. The remainder of the book varies very much. The writer quotes largely from the writings of other authorities, and leaves the reader to form his own conclusions. We would prefer the reader to have had more of the author's own experience. The illustrations are excellent and there is a good index.

**Atlas of Syphilis.** By Professor LEO and ZUMBUSCH. 31 plates. London: John Bale, Sons and Danielsson Ltd. 1922. Price 30s. net.—In this Atlas the commoner and more typical syphilitic lesions are reliably represented in a series of coloured photographs taken direct from the living subject or from actual pathological specimens. The explanatory letterpress is clear and concise. The Atlas should prove a valuable aid to students and practitioners in the identification of syphilis in the many guises which it may assume.

**Cancer: its Cause, Treatment and Prevention.** By A. T. Brand, M.D., C.M., V.D. Pp. viii., 120. London: John Bale, Sons and Danielsson Ltd. 1922. Price 8s. 6d.—This is not a new book, but is a reprint of a series of addresses delivered to the East York and North Lincoln Branch of the British Medical Association, and published in the *British Medical Journal*, *Lancet* and *Aberdeen University Press* between the years 1902 to 1910. As these addresses are all on the same subject and deal almost entirely with its etiology there is a great tendency to repetition of theories and facts in each article. The author puts forward the theory that cancer is an auto-infectious disease like syphilis and tuberculosis, and further that it arises from without the body by infection, but this infection only occurs under certain favourable conditions, such as irritation, injury or senile degeneration. He allows that it is not as infectious as are many other diseases. His argument is that all diseases which are infectious to the individual are transmissible to others and have an external origin. Now cancer is infectious to the individual, therefore cancer is transmissible to others and has an external origin. The author quotes many cases of *cancer à deux*. He emphasises the existence of cancer houses, and condemns the Ministry of Health for not making cancer notifiable. Under treatment he states that surgery is unsatisfactory, that it is more than doubtful if any operative treatment is ever successful in the complete eradication of cancer, and quotes a case where recurrence occurred in the femur nine years after amputation of the breast. He advises the use of auto-serum. He draws off 10 c.c.m. of blood from a patient's vein, allows the blood to coagulate, and reinjects ·5 c.c.m. of the serum every week under the patient's skin. No cases of cure are quoted after using this treatment. The book concludes with a quotation from Sir J. Bland Sutton on the danger of purveying the parasites of cancer in uncooked food and in water.

**Le Médecin Devant L'Assistance et L'Enseignement Psychiatriques.** By Dr. Henri Damaye. Pp. 124. Paris: A. Maloine et Fils. 1922.—This little book, while professing to be a review of the present position of psychiatry in France, is actually a plea for the better and earlier study of mental disease, particularly in hospital and military practice. According to the writer, this branch of medicine is sadly neglected in France; in fact, outside asylum practice, there is no psychiatry. Yet, in the words which stand at the head of one of his chapters, " The doctor's place is not among the incurables ; he should treat, relieve and cure."

**Aids to Organotherapy.** By IVO GEIKIE COBB, M.D., M.R.C.S. Pp. 178. London : Baillière, Tindall and Cox. 1922. Price 5s. net.—To attempt a critical review of endocrinology that shall indicate to the impatient reader what is fact and what is fancy in so small a space as that offered by this little volume is to court failure. Yet Dr. Cobb has achieved a measure of success sufficient to make the book a real help and a sound guide. There is, of course, a good deal of speculative and hypothetical matter, but so far as any book of such modest proportions can help us to understand an unlimited and indefinite subject this book does so, and may therefore be recommended to the general reader.

**The Heart as a Power Chamber.** By HARRINGTON SAINSBURY, M.D., F.R.C.P. Pp. xii., 248. London : Oxford Medical Publications. 1922. Price 12s. 6d. net.—This small book, with the sub-title, " A Contribution to Cardiodynamics," is a discursive commentary, after the manner of an eighteenth-century treatise on medicine, on the newer cardiology. That it contains much that is wise and to the point was to be expected, when its author's long association with the experimental physiology of the circulation is recalled, and a perusal of its pages does not disappoint these expectations. It is the more to be regretted that the style affected by the writer is such that few, in these utilitarian days, will have the patience to sift the book carefully in search of the nutritive matter that is there.

**The Practical Medical Series.** Vol. I. Chicago : The Year Book Publishers. 1922. Price $3.—*The Practical Medicine Series* is evidently to be to America what the *Medical Annual* is to England. Volume I. is devoted to general medicine, and gives a comprehensive view of recent work and ideas, and although most of the papers are taken from the medical literature of America, yet a considerable number of English and European papers have been referred to. The book is interesting and readable, though in places the meaning is by no means clear. It is a pity that the proofs have not been more carefully read, for the volume shows many printers' errors.

**Oto-Rhino-Laryngology.** By Dr. GEORGES LAURENS. Translated by H. CLAYTON FOX, F.R.C.S.I. Second English Edition. Pp. xii., 350. Bristol : John Wright & Sons Ltd. 1922. Price 17s. 6d. net.—The first edition was reviewed in this *Journal* for September, 1921. That now dealt with is a translation from the fourth (French) edition, which may be taken as an indication of its success. The work is essentially practical in nature and profusely illustrated. The book is designed as a guide to the general practitioner in the diagnosis and treatment of diseases of the nose, throat and ear, and can be cordially recommended. By avoiding the description of operations which lie within the scope of the specialist, he has been enabled to deal in great detail with the question of diagnosis and of non-operative and minor operative treatment. Since this book reflects current French practice, it will be found in some minor respects divergent from the English. In this edition, however, the use of the metric system, referred to in the review of the first edition, has been abandoned.

**Medical Classics Series: Selected Works of Thomas Sydenham, M.D., with a short Biography and Explanatory Notes.** By JOHN D. COMRIE, M.A., B.Sc., M.D., F.R.C.P. Ed., Lecturer on Historical Medicine and on Clinical Medicine in the University of Edinburgh. 1922. London: John Bale, Sons and Danielsson Ltd.—In this small volume Dr. Comrie has given us a charming account of Sydenham's life and friendships and about one-third of his writings, including many of those diseases which he was the first to describe and of those clinical pictures which will remain true and valuable for all time. Sydenham set to work to observe and put down the actual clinical facts of disease as perfectly as the botanist described vegetable growths, because he held that the science of the time was quite incapable of explaining the causes, in spite of the flood of unproved theories, mechanical, chemical and others, which then dominated men's thoughts, and, what was worse, their prescriptions. How ably he built this foundation these selections show most beautifully, and it is just this intensive study of clinical symptoms that we to-day have still to complete. Though science is now much more fruitful, it is still far from explaining all, and we still tend to rely on unproved theories and to neglect the observation and weighing of facts, as Mackenzie and others have pointed out. Hence the value of a study of Sydenham to-day. Dr. Comrie has added a useful bibliography of his works, which shows how widely he was admired and read throughout Europe. Though like some of our students to-day he spent four years in fighting, he had ample time afterwards at Oxford for a study of his profession, first at Magdalen Hall, then at Wadham, and as a medical fellow at All Souls', where Millington, Willis, Wren, Boyle, and others were the scientific leaders of the time. With some of these he kept up a life-long intimacy. Dr. Comrie's all too brief biography tells us, too, of his stay at Montpellier, where he seems to have been much impressed with the teaching of Barbeyrac; and of his friendship with two other men from the Bristol district, John Locke and Thomas Dover. Most of all, we value his exposition of Sydenham's methods of treatment, his reliance on the few drugs of tried value—bark, iron, liquid laudanum, and mercury—and his avoidance of the incredible mixtures then in fashion. One is, however, apt to overlook Sydenham's own brief statement of his position in the epidemic diseases at the foot of page 101. The short introductions to each treatise are just what are wanted for understanding the text, and the book as a whole is a most illuminating and scholarly introduction to this "English Hippocrates."

**Notes on Bacteriology for Dental Students.** By F. St. J. Steadman, D.P.H., L.D.S. (Eng.). Pp. 48. London: John Bale, Sons & Danielsson Ltd. 1922. Price 3s. 6d. net.—Bacteriology has been gradually taking a more and more prominent place in medical and surgical work, but until quite recently the dental surgeons and, for that matter, the dental students have viewed it as a more or less interesting subject, with little or no application to their daily work. Up to date only two books on dental bacteriology have appeared:—

(1) *The Micro-organisms of the Human Mouth* (Miller).
(2) *The Mycology of the Mouth* (Goadby).

The first was composed by an American late in the last century, and the second composed by an Englishman, and published in 1902. Much valuable and interesting work has been carried out since those days. It is more than time that a book was forthcoming dealing with the subject in all its dental aspects. The book under review contains less than fifty pages. It is therefore impossible to deal with the subject in a comprehensive manner. In the preface the author explains that these "Notes" are intended for students who are not taking a practical course in bacteriology. It is therefore a matter for regret that Chapter ii., which deals with the methods of cultivation and investigation of bacteria, has not been more fully dealt with; and further that a brief description of the methods of taking cultures from the mouth and their inoculation into suitable media has been excluded altogether. This little book of "Notes" will prove exceedingly helpful in many ways. The author has clearly and ably dealt in a few words with the subject of the production of toxins, immunity, etc. Chapter v., in which the subjects of the opsonic index, agglutination and Wassermann reaction are described, is an excellent example of how much information may be conveyed in a few words. The publication of this book will help to draw attention to the subject with which it deals, and assist in placing bacteriological science in the position it should occupy in the practice of modern dental surgery.

